# Restoration of Joint Inclination in Total Knee Arthroplasty Offers Little Improvement in Joint Kinematics in Neutrally Aligned Extremities

**DOI:** 10.3389/fbioe.2021.673275

**Published:** 2021-04-29

**Authors:** Zhi-Wei Wang, Liang Wen, Yi-Chao Luan, De-Si Ma, Xiang Dong, Cheng-Kung Cheng, Tie-Bing Qu

**Affiliations:** ^1^Department of Orthopaedics, Beijing Chaoyang Hospital, Capital Medical University, Beijing, China; ^2^School of Biological Science and Medical Engineering, Beihang University, Beijing, China; ^3^Beijing Naton Medical Technology Innovation Center Co., Ltd., Beijing, China; ^4^School of Biomedical Engineering, Shanghai Jiao Tong University, Shanghai, China; ^5^The Center of Diagnosis and Treatment for Joint Disease, China Rehabilitation Research Center, Capital Medical University, Beijing, China

**Keywords:** knee kinematics, total knee arthroplasty, joint line inclination, kinematic alignment, mechanical alignment, computational simulation

## Abstract

Kinematically aligned total knee replacements have been shown to better restore physiological kinematics than mechanical alignment and also offer good postoperative satisfaction. The purpose of this study is to evaluate the extent to which an inclined joint line in a kinematically aligned knee can alter the postoperative kinematics. A multi-body dynamic simulation was used to identify kinematic changes in the joint. To accurately compare mechanical alignment, kinematic alignment and a natural knee, a “standard” patient with neutral alignment of the lower extremities was selected for modeling from a joint database. The arthroplasty models in this study were implanted with a single conventional cruciate-retaining prosthesis. Each model was subjected to a flexion movement and the anteroposterior translation of the femoral condyles was collected for kinematic analysis. The results showed that the mechanical alignment model underwent typical paradoxical anterior translation of the femoral condyles. Incorporating an inclined joint line in the model did not prevent the paradoxical anterior translation, but a 3° varus joint line in the kinematic alignment model could reduce the peak value of this motion by about 1 mm. Moreover, the inclined joint line did not restore the motion curve back to within the range of the kinematic curve of the natural knee. The results of this study suggest that an inclined joint line, as in the kinematic alignment model, can slightly suppress paradoxical anterior translation of the femoral condyles, but cannot restore kinematic motions similar to the physiological knee. This finding implies that prostheses intended to be used for kinematic alignment should be designed to optimize knee kinematics with the intention of restoring a physiological motion curve.

## Introduction

Mechanical alignment (MA) is often considered the gold standard alignment technique for total knee arthroplasty (TKA). MA aims to restore a neutral alignment and achieve a joint line perpendicular to the mechanical axis of the lower extremities. It had been developed to reduce “malalignment,” avoid uneven mediolateral load distribution (Benjamin, 2006), and consequently reduce the risk of early implant failure (Ritter et al., 1994; Fang et al., 2009; Li et al., 2017; Vandekerckhove et al., 2017).

Although mechanically aligned total knee arthroplasty (MA-TKA) offers predictable and excellent survivorship of implants, the high rate of postoperative dissatisfaction is of concern (Robertsson et al., 2000; Beverland, 2010; Bourne et al., 2010; Nam et al., 2014), at least in part owing to changes in knee kinematics when the natural knee is replaced with a prosthesis (Li et al., 2006; Bull et al., 2008; Akbari Shandiz et al., 2016; Angerame et al., 2019). An alternative technique, kinematic alignment (KA), was introduced by Howell et al. (2008) to restore the anatomical shape of the tibio-femoral articulation (Konan et al., 2015). Kinematically aligned total knee arthroplasty (KA-TKA) has been shown to better restore knee kinematics than MA-TKA under *in vivo* radiological assessment (Howell et al., 2013), gait analysis (Blakeney et al., 2019), cadaveric studies (Keshmiri et al., 2019; Koh et al., 2019a,b; Maderbacher et al., 2019) and computer simulations (Ishikawa et al., 2015; Theodore et al., 2017). However, it is implausible that “near normal” knee kinematics can be achieved by only restoring the anatomical morphology of the tibiofemoral joint (Ishikawa et al., 2015), especially without the constraints of the anterior cruciate ligament (ACL) and menisci following KA-TKA (Blunn et al., 1991).

Given the inherent differences in the shape and morphology of the lower extremities in a population (Bellemans et al., 2012), a wide variation in the individual alignment of lower extremities would be expected following MA-TKA or KA-TKA. Differences in postoperative alignment have been reported to be related to kinematic changes in the joint (Maderbacher et al., 2016). In order to accurately compare knee kinematics between MA-TKA and KA-TKA, and against a natural knee (Johal et al., 2005), this study employed computational models because of their high sensitivity, high repeatability, and ability to eliminate confounding factors (Wang et al., 2012; Theodore et al., 2017). Moreover, this study introduces the concept of a “standard” patient, whose knee is representative of a neutral knee and a 3° varus joint line. As a consequence, the inclination of the joint line of the “standard” patient can be modified to meet the requirements of MA and KA without altering the tension in the collateral ligaments or the alignment of the lower extremities during model construction. The purpose of this study is to explore how postoperative kinematics are affected by changes in the inclination of the joint line, as in KA-TKA.

## Materials and Methods

### Anatomical Features of the “Standard” Patient

The “standard” patient was selected from a joint database termed the “Anatomical Database of the Knee Joint,” which was established by a research project funded by National Natural Science Foundation of China (NSFC, 81572180). The database includes 624 knees imaged from full-length weight-bearing radiographs and CT scans of patients diagnosed with osteoarthritis (Beijing Chaoyang Hospital Ethics Committee, approval number 2015-S-004).

**FIGURE 1:**
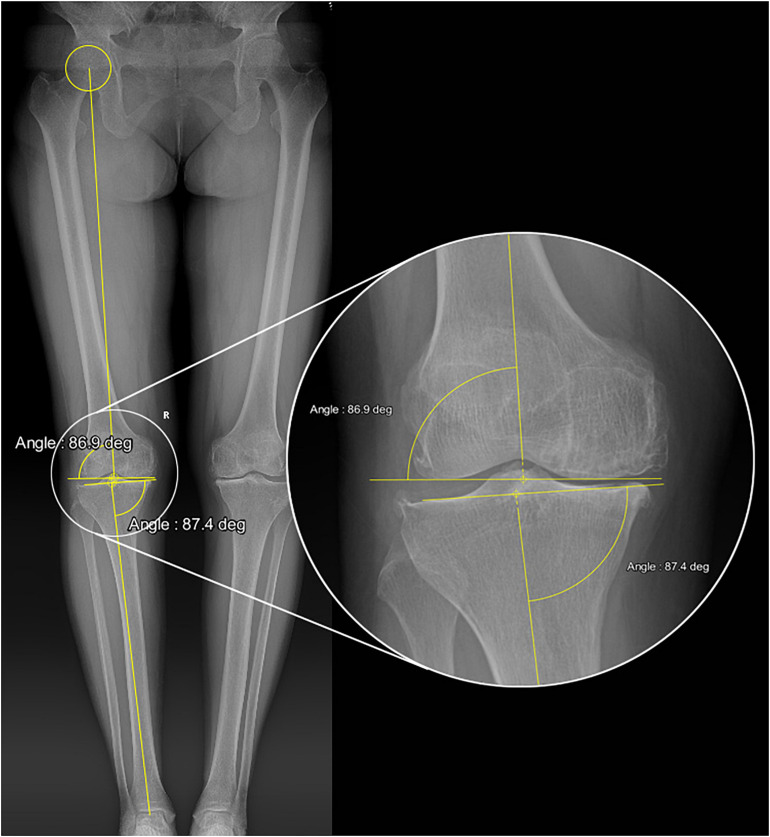
Full-length weight-bearing radiograph of the “standard” patient showing the values of mLDFA and MPTA.

The following criteria were used for selecting the “standard” patient model from the database: (i) the mechanical lateral distal femoral angle (mLDFA) and medial proximal tibial angle (MPTA) must be as close as possible to 87 degrees, which would produce postoperative neutral alignment of the lower limbs regardless of whether KA-TKA or MA-TKA was used, (ii) the angle between the surgical transepicondylar axis (TEA) and posterior condylar axis should be as close to three degrees as possible, and (iii) the medial tibial plateau’s posterior slope should be as close to seven degrees as possible. The selected “standard” subject was a female patient whose right mLDFA, MPTA and tibial posterior slope were 86.9 degrees, 87.4 degrees and 6.5 degrees, respectively (Figure 1).

### Processing of Medical Images

The raw data from full-length CT scans (scanning thickness 0.625 mm) of the “standard” patient’s knees was obtained from the Anatomical Database. Three-dimensional reconstruction of the geometry of the knee joint was conducted using Mimics 17 (Materialise NV, Leuven, Belgium), from where the solid model was imported into NX 9.0 (Siemens PLM Software, TX, United States) to measure the angle between TEA and posterior condylar axis (3.2 degrees for this patient) and to prepare the bone model.

### Setup of Multi-Body Dynamics Simulation Model

**FIGURE 2:**
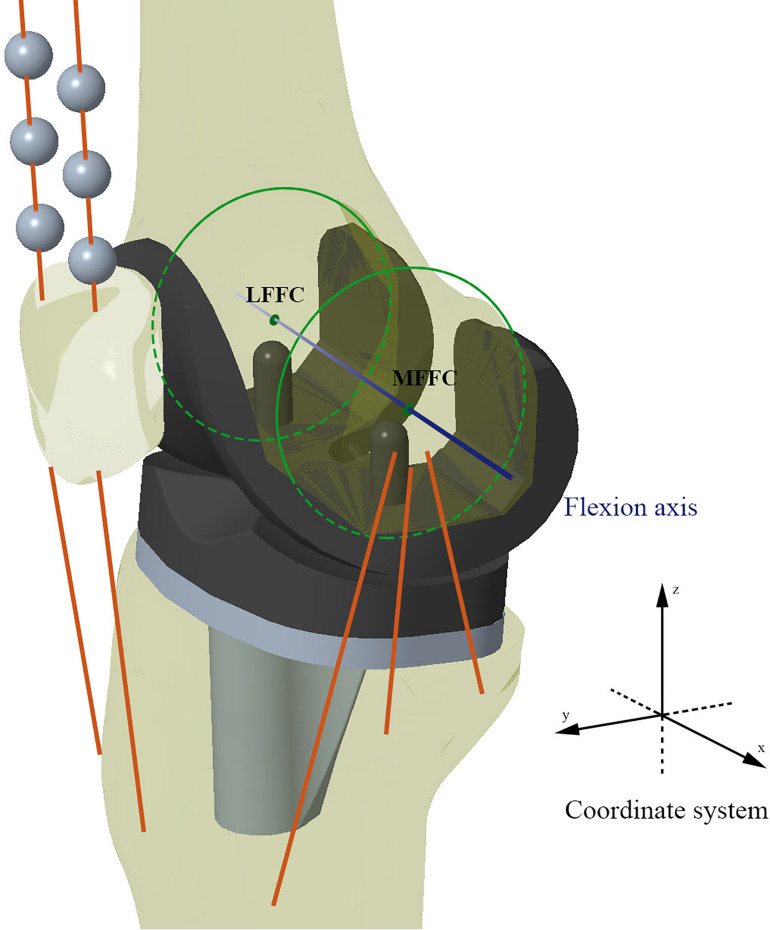
Schematic diagram of the benchmark TKA model (Model 1). MFFC and LFFC are the medial flexion facet center and the lateral flexion facet center of the corresponding femoral component’s posterior condyle, respectively. The knee flexion axis was defined as the connecting line between MFFC and LFFC.

Three-dimensional solid models of a knee prosthesis were constructed by reverse engineering retrieved fixed bearing cruciate-retaining prostheses (NexGen CR-Flex, Zimmer, Warsaw, IN, United States). First, a benchmark model (Figure 2) was constructed using the MA surgical technique (Insall et al., 1985), with the femoral and tibial bone cuts being performed separately using the measured resection method (Daines and Dennis, 2014). On the coronal plane, the distal femoral resection and proximal tibial resection were performed orthogonal to the femoral and tibial mechanical axes, respectively. The femoral mechanical axis was defined by a line connecting the center of the intercondylar notch and the center of the femoral head, while the tibial mechanical axis was defined by a line connecting the center of the talus and the midpoint of a line between the posterior cruciate ligament (PCL) insertion and medial third of the tibial tubercle. Rotation of the femoral component was determined by the orientation of the TEA, and the tibial component was implanted with a posterior slope of 7°.

The femoral component and tibial component were assembled by placing the most distal points of the femoral condyles on the lowest points of the polyethylene tibial insert. The medial and lateral flexion facet centers (FFC) were generated by circular fitting of the corresponding posterior condyles of the femoral component (Iwaki et al., 2000). The line connecting the medial and lateral FFC was designated as the flexion axis (*x* axis), the mechanical axis of the lower extremity was designated as the *z*-axis, and the *y*-axis was automatically generated at the intersection of the *x*-axis and *z*-axis. The *y*-axis was taken as the reference direction for recording femoral anteroposterior translation. A Cartesian coordinate system was created for both the tibial and femoral components in this original position (Grood and Suntay, 1983; Figure 2).

The software ADAMS_View (MSC Software, CA, United States) was used to construct the dynamic models and for kinematic simulations. The density of the tibia, femur, patella and prosthetic components was set as 0.8. Rigid beads with a density of 0.1 were created to simulate the quadriceps wrapping around the trochlear groove. The contact interface of each solid element in the model was “solid to solid” and the friction coefficients of tibiofemoral articulation and patellofemoral articulation were set as 0.04 and 0, respectively (Godest et al., 2002). A ground reaction force (1.5 × body weight = 750 N) was imposed on the centroid of the tibial component to simulate gravity (D’Lima et al., 2007). The femoral component was only permitted to rotate in flexion around the *x*-axis, while the tibial component was only constrained in flexion–extension and unconfined in all other directions. The patellar component and rigid beads were not constrained in any direction.

The force elements of the medial collateral ligament (MCL), lateral collateral ligament (LCL), PCL, patellar tendon, quadriceps, hamstrings and biceps were incorporated into the models. The origin and insertion points of the various ligaments were referenced from literature (Edwards et al., 2007; LaPrade et al., 2007; Yan et al., 2010). The MCL was considered as an anterior, oblique and deep fiber bundle, the PCL was considered as an anterior and a posterior fiber bundle, and the LCL was considered as a single fiber bundle (Abdel-Rahman and Hefzy, 1998). All bundles of ligaments were simulated as nonlinear force elements by the following constitutive equation:

$$F_{j}=\left\{ \begin{array}{c} 0;\varepsilon_{\text{j}}\leq 0\\ K\,1_{j}\,{\left(L_{j}-L_{0\,j}\right)}^{2};0<\varepsilon_{\text{j}}\leq 2\,\varepsilon_{1}\\ K\,2_{j}\,\left[L_{j}-\left(1+\varepsilon_{1}\right)\,L_{0\,j}\right];2\,\varepsilon_{1}<\varepsilon_{j} \end{array}\right.$$

Where *F* is the tensile force of the element, ε*_*j*_* is the strain in the *j*th element, K1*_*j*_* and K2*_*j*_* are the stiffness coefficients of the *j*th spring element for the parabolic and linear regions, respectively, and L*_*j*_* and L0*_*j*_* are its current and slack lengths, respectively. The linear range threshold was ε*_1_* = 0.03. The stiffness coefficients of each fiber bundle are shown in Table 1.

**TABLE 1:** The stiffness coefficients of the ligaments in current models.

	K1 (N/mm)	K2 (N/mm)
MCL-anterior	10.00	91.25
MCL-oblique	5.00	27.86
MCL-deep	5.00	21.07
PCL-anterior	31.26	125.00
PCL-posterior	19.29	60.00
LCL	10.00	72.22

*MCL, medial collateral ligament; PCL, posterior collateral ligament; LCL, lateral collateral ligament.*

Both the quadriceps and patellar tendons were designated as medial and lateral fiber bundles and simulated as tensile spring elements (Piazza and Delp, 2001) with stiffness coefficients of 2,000 N/mm (Yu et al., 2001) and 1,142 N/mm (Hashemi et al., 2005), respectively. The biceps femoris and hamstring were designated as single bundles and simulated as tensile spring elements.

The total simulation time was 420 s, with the first 300 s being allowed for the model to reach mechanical equilibrium before initiating the flexion movement. The femoral component was flexed from 0 to 120° at an angular velocity of 1 degree/s.

### Model Validation

**FIGURE 3:**
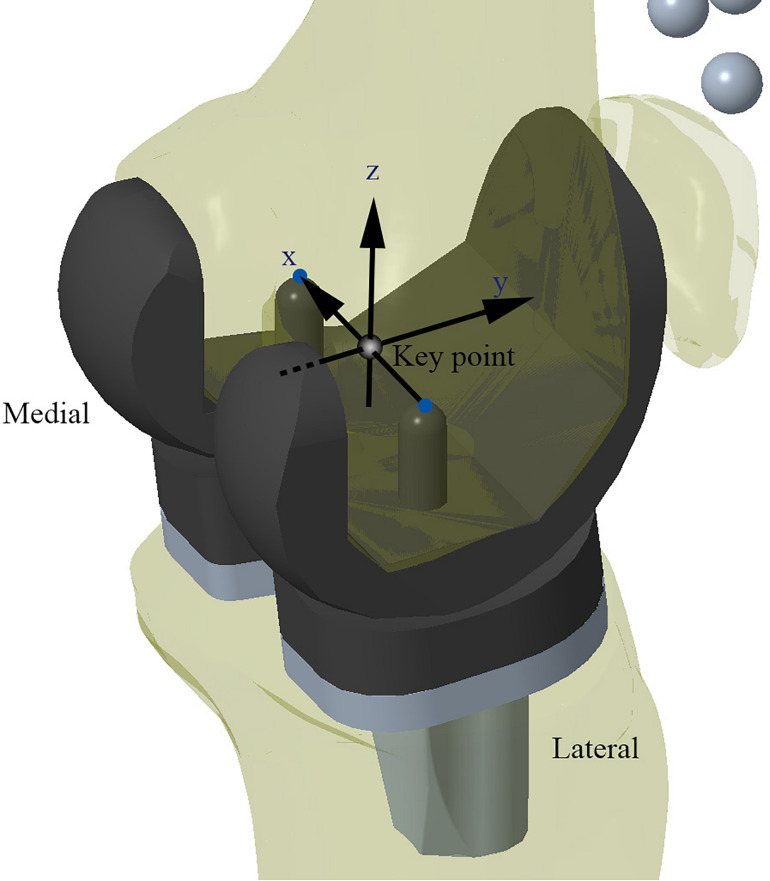
Schematic diagram of the Model 1 for validation. Kinematic data recorded from the “Key point” marker was used for model validation.

In this study, the TKA models were built using a method previously validated by our research team (Wang et al., 2012; Zhao et al., 2015). Moreover, the benchmark model was validated against the results of an *in vivo* kinematic study using the same implants and alignment technique (Suggs et al., 2009). As with the *in vivo* study, the midpoint between the peg tips of the femoral component was designated as the “Key point” in the benchmark model (Figure 3). Femoral translation along the *y*-axis and tibial rotation around the *z*-axis were determined from this “Key point” and compared with Suggs’ *in vivo* study.

### Adjustment of Joint Line Inclination and Establishment of KA-TKA Model

**FIGURE 4:**
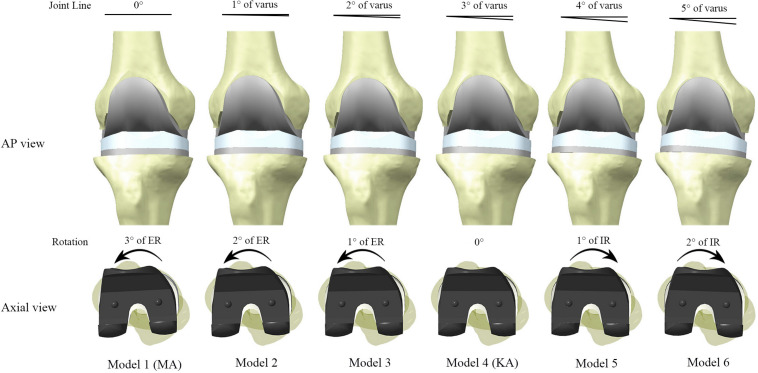
Models constructed in this study with different joint alignment. The joint line inclination and internal rotation of the implants were increased in 1° increments between models, up to Model 6 (5° varus of joint line, 2° internal rotation of implant). Model 4 reproduced the original tibiofemoral articular geometry of the “standard” patient, and therefore met the technical requirements of kinematic alignment (KA). ER and IR represent internal rotation and external rotation relative to the posterior condylar axis, respectively.

Once the benchmark model (Model 1) had been validated, the inclination of the joint line was adjusted to produce six unique models, but the relative position of the femoral component to the tibial component remained consistent across all models. The algorithm used required that for every 1° of varus rotation of the implants around the *y*-axis, the implants simultaneously underwent 1° of internal rotation around the *z*-axis. This algorithm theoretically would not cause abnormal tension in the collateral ligaments throughout the full range of motion of the knee joint. After five successive adjustments, six models were constructed (Figure 4), in which Model four met the requirements of KA with 3° of varus rotation of the joint line and 3° of internal rotation relative to TEA, which is consistent with the articular morphology of the “standard” patient.

### Kinematic Analysis

The translation of the medial and lateral FFC along the *y* axis was recorded from 0 to 120° of flexion. To allow direct comparison between models, the coordinates of the FFC in all models was consistent with those in the benchmark model.

For the kinematic evaluation of different alignment techniques, the distances between the FFCs and the ipsilateral posterior edge of the polyethylene insert were collected after every 10° of flexion in Model 1 and Model 4, and the corresponding data were analyzed against *in vivo* data (Johal et al., 2005).

The post-processing module in ADAMS (MSC Software, CA, United States) was used for the kinematic analysis, and Microsoft Excel (version 2013, Microsoft, Redmond WA, United States) was used for data visualization.

## Results

### Model Validation

**FIGURE 5:**
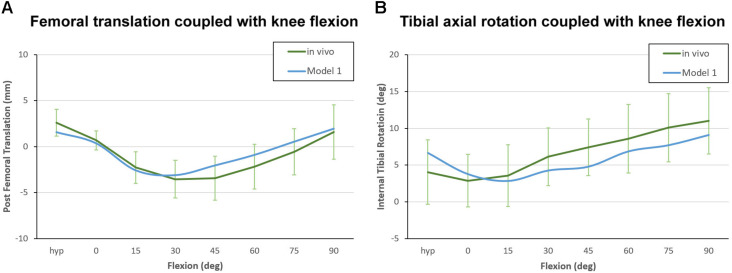
Kinematic curve of Model 1 plotted against *in vivo* data for model validation. **(A)** Femoral translation coupled with knee flexion, with positive values indicating posterior translation relative to the reference position. **(B)** Tibial axial rotation coupled with knee flexion, with positive values indicating internal tibial rotation relative to the femur.

The kinematic curves for anteroposterior translation showed a similar trend between the benchmark model and *in vivo* study. The maximum paradoxical anterior translation of the femur occurred at 45° of flexion (3.4 ± 2.3 mm) in the *in vivo* study, while the maximum anterior translation in benchmark model occurred at 30° of flexion (3.1 mm). Throughout the flexion cycle, the difference in femoral translation between the benchmark model and *in vivo* study did not exceed 1.5 mm (Figure 5A). The internal tibial rotation curves for the *in vivo* study and benchmark model also showed similar trends, and both curves showed a certain degree of reverse rotation in the early stages of knee flexion, but the reverse rotation of the benchmark model lasted slightly longer (Figure 5B).

### Impact of Joint Line Inclination on Postoperative Kinematics

After the models were preloaded with gravity and reached mechanical equilibrium, the medial and lateral FFCs were found to have moved backward by an average distance of 1.6 and 4.5 mm, respectively. Although the varus orientation of the joint line could not fully eliminate the posterior shifting of the FFCs, it did suppress the movement of the lateral FFC to a certain extent.

**FIGURE 6:**
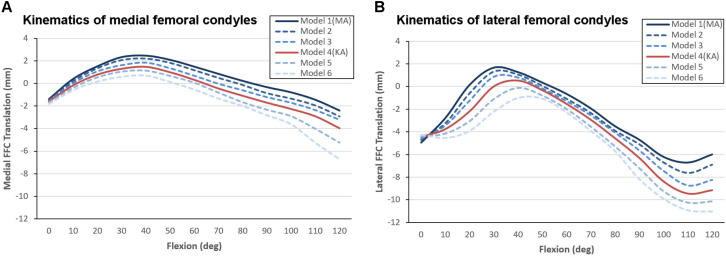
Kinematic comparison of different joint line inclinations showing anterioposterior translation of the medial **(A)** and lateral **(B)** FFCs coupled with knee flexion. The “0” value on the vertical axis represents the initial position of the model before mechanical equilibrium, positive values indicate forward positioning of the FFCs, and negative values indicate backward positioning of the FFCs.

The anteroposterior translation of the medial and lateral FFCs in all dynamic models are shown in Figure 6. All models showed paradoxical anterior translation of the medial FFC (Figure 6A) and lateral FFC (Figure 6B) in early flexion, but the amplitude was suppressed in the models with a more inclined joint line. Taking Model 1 (MA-TKA) and Model 4 (KA-TKA) for example, the paradoxical anterior translation of the medial FFC in the KA-TKA model was 1mm less than in the MA-TKA model at 30° of flexion, while the anterior translation of the lateral FFC in KA-TKA was 1.2 mm less than in MA-TKA. In addition, the peak anterior translation of the lateral FFC in the KA-TKA model was delayed by about 10° of flexion in comparison to the MA-TKA model. Although placing the joint line in a varus position (Model 2–6) could suppress the paradoxical anterior movements, it did not modify the shape of the motion curves (Figures 6A,B).

### Comparative Kinematic Analysis Against the Natural Knee

**FIGURE 7:**
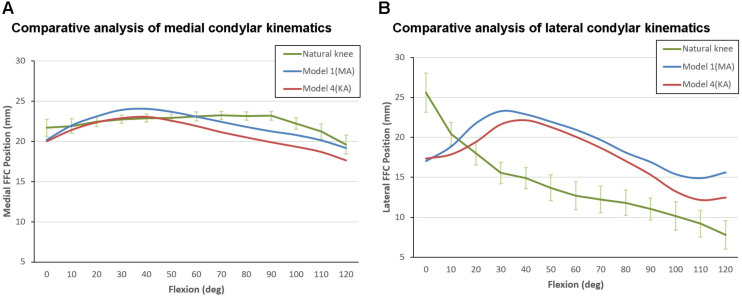
Kinematic comparison between Model 1 (MA-TKA), Model 4 (KA-TKA) and the “living” knee (Johal et al., 2005). The motion curves of the medial FFCs **(A)** and lateral FFCs **(B)** were compared. The values on the vertical axis indicate the distance between the FFCs and the ipsilateral posterior edge of tibia condyles (for the natural knee), or the distance between the FFCs and the ipsilateral posterior edge of the polyethylene insert (for the models).

The motion curves from the TKA models were found to be considerably different from that of the natural knee, regardless of the inclination of the joint line (Figure 7). The initial positions of the medial FFCs from the MA-TKA and KA-TKA models were 1.5 and 1.7 mm further back from the position of medial FFC of the natural knee in full extension (Johal et al., 2005), while the initial positions of the lateral FFCs were 8.6 and 8.2 mm behind that of the natural knee, respectively. Although the medial FFCs of the MA-TKA and KA-TKA models showed characteristic paradoxical anterior translation, and a large part of the corresponding motion curves exceeded the 95% confidence interval of that of the natural knee, the difference in positioning of the medial FFCs between the TKA models and natural knee was not so obvious (Figure 7A). In contrast, the motion curves of the lateral FFCs were obviously different from that of the natural knee for both MA and KA models (Figure 7B). The greatest difference in the position of the lateral FFCs position between MA-TKA and the natural knee occurred at 30° of flexion (7.7 mm), while that between KA-TKA and the natural knee occurred at 40° of flexion (7.2 mm).

## Discussion

This study found that when an implanted knee is placed in neutral alignment, having a varus joint line (KA-TKA) can better suppress paradoxical anterior translation of the femoral condyle than an orthogonal joint line (MA-TKA). However, the motion patterns on kinematic curves for both are still obviously different from that of the natural knee. This finding suggests that reproducing a joint line inclination similar to that of the natural knee does not bring considerable kinematic advantages to neutrally aligned lower limbs.

The advantage of using a “standard” patient for computational modeling is that it is not necessary to modify the position and properties of bony elements and soft tissue constraints when adjusting the joint line inclination. In other words, only this “standard” patient can meet the surgical requirements of MA-TKA and KA-TKA while maintaining a neutral alignment of the lower extremities and constant initial position of the ligament constraints during modeling. Using this concept of a “standard” patient allows subtle kinematic differences between the two alignment techniques to be visualized. Although only a small fraction of the population would meet the criteria of such a “standard” patient (Hirschmann et al., 2019), using this method allows for a direct comparison between the three kinematic scenarios mentioned above; MA-TKA, KA-TKA and natural knee.

Postoperative studies have reported abnormal kinematics after TKA to be correlated with patient dissatisfaction, poor clinical outcomes and increased implant wear (Dhurve et al., 2017; Vandekerckhove et al., 2017; Angerame et al., 2019). Therefore, considerable resources have been put towards improving surgical techniques and prosthetic designs with the aim of optimizing joint kinematics after TKA (Howell et al., 2013; Digennaro et al., 2014; Akbari Shandiz et al., 2016; Roth et al., 2018).

From the surgical technique perspective, KA-TKA procedures have shown promising clinical outcomes that are at least not inferior to those of MA-TKA (Young et al., 2016; Shelton et al., 2017, 2019; Howell et al., 2018; Maderbacher et al., 2019). The results of this current study showed that KA could slightly suppress the paradoxical anterior translation of the femur, which was consistent with previous studies (Ishikawa et al., 2015; Maderbacher et al., 2019). Thus, it is anticipated that more considerable improvements would be difficult when performing KA-TKA in a neutrally aligned knee, especially when using a conventional CR prosthesis. One reason might be that the “standard” patient represents the ideal candidate with excellent knee kinematics after MA-TKA and, regardless of whether the KA or MA technique is used, the soft tissue constraints around the knee do not need to be released, which maintains a good ligament balance after TKA.

From the prosthetic design perspective, a NexGen CR-Flex prosthesis was chosen for all TKA models in this study because *in vivo* data was available for model validation (Suggs et al., 2009). However, the design features of the NexGen prosthesis are not conducive to replicating physiological kinematics, including G-curves on the femoral component where the radius of curvature varies widely and a symmetrical insert with low-conformity shallow concavity which allows unusual anteroposterior movement of the femoral condyles during flexion. This study focused on kinematic changes caused by the inclination of the joint line, while other confounding factors were not considered, such as different designs, alignment of the lower extremities, ligament releasing, etc.

In this study, after mechanical equilibrium had been reached, the position of the femur was found to be slightly posterior to the tibia, and this phenomenon was more obvious for the lateral femoral condyle. Posterior positioning of the femur is not only related to the posterior slope of the tibial component, but also to a laxity or weakness in the ACL (Beynnon et al., 2002). The tension in the ACL has also been shown to play an important role in the “screw-home” motion pattern at the end of knee extension (Moglo and Shirazi-Adl, 2005). When performing TKA using a conventional CR prosthesis, the posterior positioning of the femur due to the loss of the ACL makes it impossible to replicate the “screw-home” motion of the natural knee. Therefore, the paradoxical anterior translation of the femur observed in this study could be understood as the process of the femur returning from an abnormal initial posterior position to a relatively normal forward position.

The results of this study imply that MA-TKA alone may not be the sole cause of kinematic abnormalities observed when using this surgical technique, even though previous studies speculated that MA may be directly related to patient dissatisfaction (Nam et al., 2014; Delport et al., 2015; Ishikawa et al., 2015; Hutt et al.,2016a,b; Theodore et al., 2017; Vendittoli and Blakeney, 2017; Blakeney et al., 2019; Koh et al., 2019a; Maderbacher et al., 2019). The better clinical outcomes following KA-TKA may be related to other factors, such as less soft tissue release (An et al., 2019). In contrast, it has been reported that the proportion of MA-TKAs requiring a ≥2 mm ligament release was 34 and 30% for the medial and LCL, respectively (Gu et al., 2014).

In this study, the TKA model was validated against *in vivo* data using the same prosthesis and surgical technique, while most previous studies were validated against cadaveric models or virtual test platforms (Ishikawa et al., 2015; Theodore et al., 2017; Roth et al., 2018; Koh et al., 2019b; Maderbacher et al., 2019). To allow for this comparison against *in vivo* data, the initial length of the PCL elements had to be extended to fit the kinematic curve from the *in vivo* study (Suggs et al., 2009). The reason for such adjustment is that dynamic modeling is routinely conducted with the knee in extension when the PCL is relaxed and slack.

There are some limitations to this study that should be noted. First, the simulation did not consider the kinematics of the patellofemoral joint because the different methodologies used for patellar tracking make a comparison between studies difficult (Katchburian et al., 2003). Moreover, KA theoretically does not present any particular advantages for patellofemoral kinematics. Second, only the anteroposterior translation of the femur relative to the tibia was analyzed instead of all six degrees of freedom, and only one motion, deep bending, was simulated. This set-up was to facilitate the kinematic comparison (i) among the models, (ii) against the kinematic data from an *in vivo* study (Suggs et al., 2009) and (iii) against the kinematic curve from a “living” knee (Johal et al., 2005). The kinematics of the knee joint can show considerable variation between different daily activities (Kozanek et al., 2009; Li et al., 2013). Among them, the motion pattern of the weight-bearing squat or lunge is the most consistent, going through a medial pivot movement (Angerame et al., 2019). The medial pivot motion has been extensively evaluated in both cadaver studies (Iwaki et al., 2000) and in living knees (Johal et al., 2005). Therefore, the models in this study only simulated weight-bearing deep flexion. Third, the models in this study used a single commercial CR prosthesis, so the kinematic results may not be representative of all prostheses on the market. Finally, the knee models were flexed from 0 to 120° and did not proceed into a “high flexion” situation which exceeded 120°, which has been described as the “passive deep flexion arc” in literature (Williams and Logan, 2004). Beyond 120°, gravity and the pushing effect of soft tissues behind the knee joint are gradually imposed on the proximal tibia, and the femoral condyles continue to move backward to accomplish a greater degree of flexion. Since no quantified *in vivo* data of this pushing force was available from literature, this study only considered knee flexion up to 120°. Future studies should consider a wider variety of knee joints with different constitutional deformities, and the movements of the natural knees should be presented and analyzed according to the alignment status of the natural knee.

## Conclusion

In a neutrally aligned knee joint, the inclined joint line in KA-TKA has little kinematic superiority over the orthogonal joint line in MA-TKA. This study suggests that kinematic optimization should be incorporated into the design of KA specific prostheses. The results also showed that the KA technique may be more suitable for patients with constitutional varus or valgus knees.

## Data Availability Statement

The original contributions presented in the study are included in the article/supplementary material, further inquiries can be directed to the corresponding author/s.

## Ethics Statement

The studies involving human participants were reviewed and approved by the Beijing Chaoyang Hospital Ethics Committee. The patients/participants provided their written informed consent to participate in this study. Written informed consent was obtained from the individual(s) for the publication of any potentially identifiable images or data included in this article.

## Author Contributions

Z-WW, LW, and T-BQ contributed to the study design, validation of the simulation models, and interpretation of simulation results and drafted the manuscript. D-SM contributed to the extraction of kinematic data and data visualization. Y-CL and XD built and debugged the simulation models. C-KC contributed to the interpretation of simulation data and software support. All authors read and approved the final manuscript.

## Conflict of Interest

XD was employed by the company “Beijing Naton Medical Technology Innovation Center Co., Ltd.” The remaining authors declare that the research was conducted in the absence of any commercial or financial relationships that could be construed as a potential conflict of interest.

## Abbreviations

MA: mechanical alignment
TKA: total knee arthroplasty
MA-TKA: mechanically aligned total knee alignment
KA: kinematic alignment
KA-TKA: kinematically aligned total knee arthroplasty
ACL: anterior cruciate ligament
mLDFA: mechanical lateral distal femoral angle
MPTA: medial proximal tibial angle
TEA: surgical transepicondylar axis
FFC: flexion facet center
MCL: medial collateral ligament
LCL: lateral collateral ligament
PCL: posterior cruciate ligament.
